# The Characteristics of Extreme Erosion Events in a Small Mountainous Watershed

**DOI:** 10.1371/journal.pone.0076610

**Published:** 2013-10-11

**Authors:** Nu-Fang Fang, Zhi-Hua Shi, Ben-Jiang Yue, Ling Wang

**Affiliations:** 1 State Key Laboratory of Soil Erosion and Dryland Farming on the Loess Plateau, Northwest A & F University, Yangling, PR China; 2 Institute of Soil and Water Conservation of Chinese Academy of Sciences and Ministry of Water Resources, Yangling, PR China; 3 College of Resources and Environment, Huazhong Agricultural University, Wuhan, China; Plymouth University, United Kingdom

## Abstract

A large amount of soil loss is caused by a small number of extreme events that are mainly responsible for the time compression of geomorphic processes. The aim of this study was to analyze suspended sediment transport during extreme erosion events in a mountainous watershed. Field measurements were conducted in Wangjiaqiao, a small agricultural watershed (16.7 km^2^) in the Three Gorges Area (TGA) of China. Continuous records were used to analyze suspended sediment transport regimes and assess the sediment loads of 205 rainfall–runoff events during a period of 16 hydrological years (1989–2004). Extreme events were defined as the largest events, ranked in order of their absolute magnitude (representing the 95^th^ percentile). Ten extreme erosion events from 205 erosion events, representing 83.8% of the total suspended sediment load, were selected for study. The results of canonical discriminant analysis indicated that extreme erosion events are characterized by high maximum flood-suspended sediment concentrations, high runoff coefficients, and high flood peak discharge, which could possibly be explained by the transport of deposited sediment within the stream bed during previous events or bank collapses.

## Introduction

Soil erosion poses a serious problem for sustainable agriculture and the environment [1], [2]. The soil and water conservationist is typically more interested in flood or erosion damage caused by large events than in the damage caused by smaller, more common hydrological events. Many studies have clearly emphasized that large-magnitude, low-frequency events are assumed to be dominant with respect to soil erosion [3]–[9]. Estimation of the role of sediment transport produced by extreme events is necessary for the calculation of sediment yields from basins, as a single event may represent the transport of several “normal” years [10]. However, soil erosion is a temporally compressed process; the effect of one event must be isolated when designing erosion control technologies and conservation planning [11], [12].

Previous projects have documented the effects of extreme events on total soil erosion and sediment transport on catchment scales [13]–[17]. These studies have mainly focused on extreme rainfall events and have discussed erosion based on those events. Analysis of the relationships between suspended sediment transport and rainfall characteristics during erosion events can help in understanding the factors and processes that determine sediment responses [18], [19]. However, runoff and erosion processes are strongly affected by many other factors in addition to rainfall [20]–[22], and few systematic attempts have been made to distinguish extreme rainfall events and erosion events.

**Figure 1:**
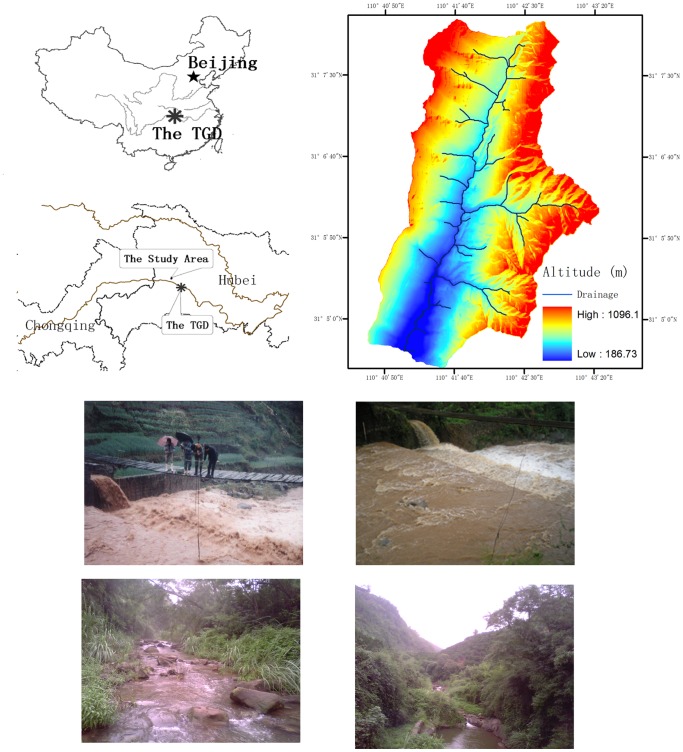
Location of the study watershed in the Three Gorges Area, China. Photos are of an overall view, the main channel, and the outlet of the Wangjiaqiao watershed. Photos are of an overall view, the main channel, and the outlet of the Wangjiaqiao watershed during an extreme event.

The Three Gorges Project (TGP) on the Yangtze River in China is the world’s largest hydropower project. Following construction of the Three Gorges Dam, many farmers resettled in surrounding mountain areas and cultivated marginal lands, which are mostly on steep slopes with soil of poor structure. The TGP is controversial for several reasons, including the likely impact of sedimentation on the operation and useful life of the reservoir [23], [24]. The TGA refers to the riparian counties along the Yangtze valley between Yichang and Chongqing (Fig. 1). This area is periodically impacted by catastrophic floods, and soil erosion is a major environmental problem [25]. High levels of suspended sediment may result in high sediment deposition rates, thereby reducing the useful life of the Three Gorges Reservoir. Thus, understanding the temporal variations of suspended sediment transport and sediment loads during extreme events is essential for future watershed management plans.

In this study, we investigated the runoff and erosion processes of the Wangjiaqiao watershed, which is a representative watershed within the TGA [26], [27]. The main objectives of this work were to (1) characterize extreme erosion events and the hydrological and sediment responses in a small watershed in the TGA and (2) improve our understanding of the factors that control sediment transport patterns and loads during extreme erosion events.

## Study Area and Methods

### Study Area

The study was conducted in the Wangjiaqiao watershed (31°5′N–31°9′N, 110°40′E–110°43′E), which lies in Zigui County in Hubei Province, China. It is approximately 50 km northwest of the Three Gorges Dam and covers an area of 16.7 km^2^ (Fig. 1). Elevations within the watershed range from 184 to 1,180 m and slopes range from 2° to 58°, with an average slope of 23°. The parent materials of this area are mostly Cretaceous and Tertiary purple shale, sandy shale, and sandstone, which contain large quantities of iron and manganese oxides in specific mineralogical forms. Two main soil great groups occur in the study watershed: purple soils derived from purple sandy shale and paddy soils developed from the purple soil. According to the USDA Soil Taxonomy, purple soils and paddy soils are classified as entisols and aquepts, respectively. The climate is subtropical, with mean temperatures between 11°C and 18°C. The annual precipitation averages 1,016 mm, 70% of which occurs between May and September. Land use is mainly a function of elevation and topography. Remnant forest patches exist primarily on steep, inaccessible peaks and slopes. Little natural vegetation is observed, and most areas are covered by secondary vegetation under human influence. A field survey was carried out in 1995 using a topographic map (scale 1∶10,000) and aerial photos. The results indicated that forest covered 44.5% of the study area, whereas cropland covered 23.3% (389.4 ha) and paddy fields covered 19.8% (330.7 ha). The other land use types were relatively minor and consisted of shrubland (3.2%), orchards (4.2%), rural residential land (3.9%), and water bodies (0.7%). The main agricultural crops are rice (*Oryza sativa* L.), maize (*Zea mays* L.), and wheat (*Triticum aestivum* L.). The streams in this watershed have a pinnate drainage pattern, and the length of the main channel is approximately 6,500 m [26].

### Field and Lab Methods

Suspended sediment yields represent the sum of the erosion produced by all active sources within a watershed, although yields cannot be used as reliable indicators of total hill slope erosion due to the difficulty of quantifying sediment storage and identifying the mixture of sources [3]. A set of instruments consisting of a continuous recording rain gauge, water-level stage recorder, and bottle-type silt sampler were used to record rainfall, stream flow, and sediment load, respectively. The water stage was measured every 15 min and transformed into discharge using a calibrated rating curve obtained from periodic flow measurements. Suspended sediment concentrations (SSCs) were determined by the gravimetric method. Water samples were vacuum filtered through a 0.45-µm filter, and the residue was oven dried at 105°C for 24 h. Suspended sediment samples were taken only during rainfall–runoff events, and more than 10 samples were required during each event based on the “Technical code of practice on water and soil conservation monitoring” [28]. In practice, samples were collected based on the variation of the discharge and the magnitude of the SSC. Generally, samples were collected frequently during events with high SSC values and were collected infrequently during events with relatively small SSC values. The weight of each dried sample of residue and the sample volume were used to determine the SSC (g m^−3^). The suspended sediment load was then calculated from the SSC and water discharge data. Watershed runoff and rainfall data have been collected since 1989. No specific permissions were required for these sampling activities because the location is not privately-owned or protected in any way and the field activities did not involve endangered or protected species.

### Data Processes

Hydrograph separation was conducted for 205 events during the 1989–2004 period, for which we have fairly complete records of sediment concentrations or hydrographs. Floods were identified in cases in which the increase in stream discharge exceeded 1.5 times the base flow recorded at the beginning of the rainfall event [29], [30]. Runoff was separated between storm flow and base flow using the classical hydrograph separation method of Hewlett and Hibbert (1967) [31]. Some continuous events were excluded because they were hard to separate as “an event” using our separation method. Some other events with complete hydrograph records were included even though their SSC values were below the threshold for our monitoring method. Total suspended sediment load of an event is the accumulation of sediment yield during each time step. The combined SS load of the 205 events represented 68% of the total suspended sediment load during the study years.

### Statistical Analysis

The flood events were characterized using several variables (Table 1).
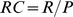


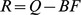
where *RC*, *R*, *P*, *Q*, and *BF* are the runoff coefficient, surface runoff, total discharge, precipitation, and base flow, respectively.

**Table 1:** Flood variables and associated abbreviations used in the statistical analysis of the rainfall–runoff-suspended sediment transport relations.

Rainfall-related variables	Runoff-related variables	Suspended sediment-related variables
Total precipitation	Runoff	Maximum flood suspended sediment concentration
(*P*, mm)	(*R*)	(*SSC_max_*, g m^−3^)
Duration of the event	Runoff coefficient	Total suspended sediment load
(*D*, h)	(*RC*)	(*TL*, t)
Maximum 30-min rainfall intensity	Flood peak discharge	
(*I_30_*, mm)	(*Q_max_*, m^3^ s^−1^)	
Antecedent precipitation one day before		
(*AP1d*, mm)		
Antecedent precipitation three days before		
(*AP3d*, mm)		
Antecedent precipitation seven days before		
(*AP7d*, mm)		



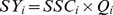


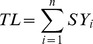


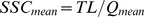
Where *SY_i_*, *SSC_i_* and *Q_i_* are the suspended sediment yield, Suspended sediment concentrations, and discharge during a time term *i*. *TL*, *Q_mean,_ and SSC_mean_* are the total suspended sediment load, mean discharge, and mean flood-suspended sediment concentration, respectively.

After evaluating the descriptive statistics, canonical discriminant analysis (CDA) was performed with the SPSS13.0 statistical software package using the variables mentioned above.

## Results

### Events Characteristics and Arrangement

An extreme event was defined classically as a rare, low-probability event, typically in relation to the exceedance of certain threshold values (e.g., means, percentiles). For this study, extreme erosion events are events that cause large amounts of SS load; no quantitative definition, e.g., in kg, was used. A partial definition might refer to an SS amount of a different order of magnitude than that caused by normal events. The definition of an extreme event in terms of damage caused by the event is discussed in [32]. A total of 10 events (approximately representing the 95^th^ percentile) were identified as extreme erosion events based on the SS load caused by those 10 events having been clearly greater than that caused by the other events.

Based on the ranking of SS loads, 205 events were classified into three regimes; extreme erosion events, normal events, and no-SS load events. Four variables were compared: *P*, *D*, *I_30_*, and *TL* (Table 2). An extreme erosion event has a destructive effect on the soil surface. The mean *SS* load of the extreme erosion events that occurred during the study period was almost 68 times that of normal events, and the mean *P* and mean *I_30_* were 2.5 and 2.1 times greater, respectively. Non-SS load events had observable hydrograph processes with a mean P of 19.6 mm but did not produce *SS* loads or their SSCs were below the threshold of our monitoring method. These results emphasize the importance of the rainfall type as a major cause of erosion. From this standpoint, the rainfall depth is an important factor in the degree of soil erosion in the study area. Meanwhile, the variance of *TL* is much higher than the variance of *P* for both extreme erosion events and normal events, which confirms the complex nature of sediment response in the study area.

**Table 2:** Statistical features of different event regimes.

Regimes	Variables	Mean	SD	Variation	Frequency(time)
	P (mm)	82.8	36.9	45%	10
I	I_30_ (mm)	15.4	7.0	46%	
	D (min)	1,408.3	899.0	64%	
	TL (kg)	7,261,812	11,308,672	156%	
	P (mm)	33.2	19.5	59%	132
II	I_30_ (mm)	7.4	7.0	95%	
	D (min)	1,034.7	776.8	75%	
	TL (kg)	106,207	179,681	169%	
	P (mm)	19.6	9.9	51%	63
III	I30 (mm)	4.8	5.2	108%	
	D (min)	834.3	583.2	70%	
	TL (kg)	0.0	0.0	/	

*P*, *D*, and *I_30_* represent precipitation depth, duration, and maximum 30-min intensity, respectively.

### Characterization of Extreme Erosion Events

**Figure 2:**
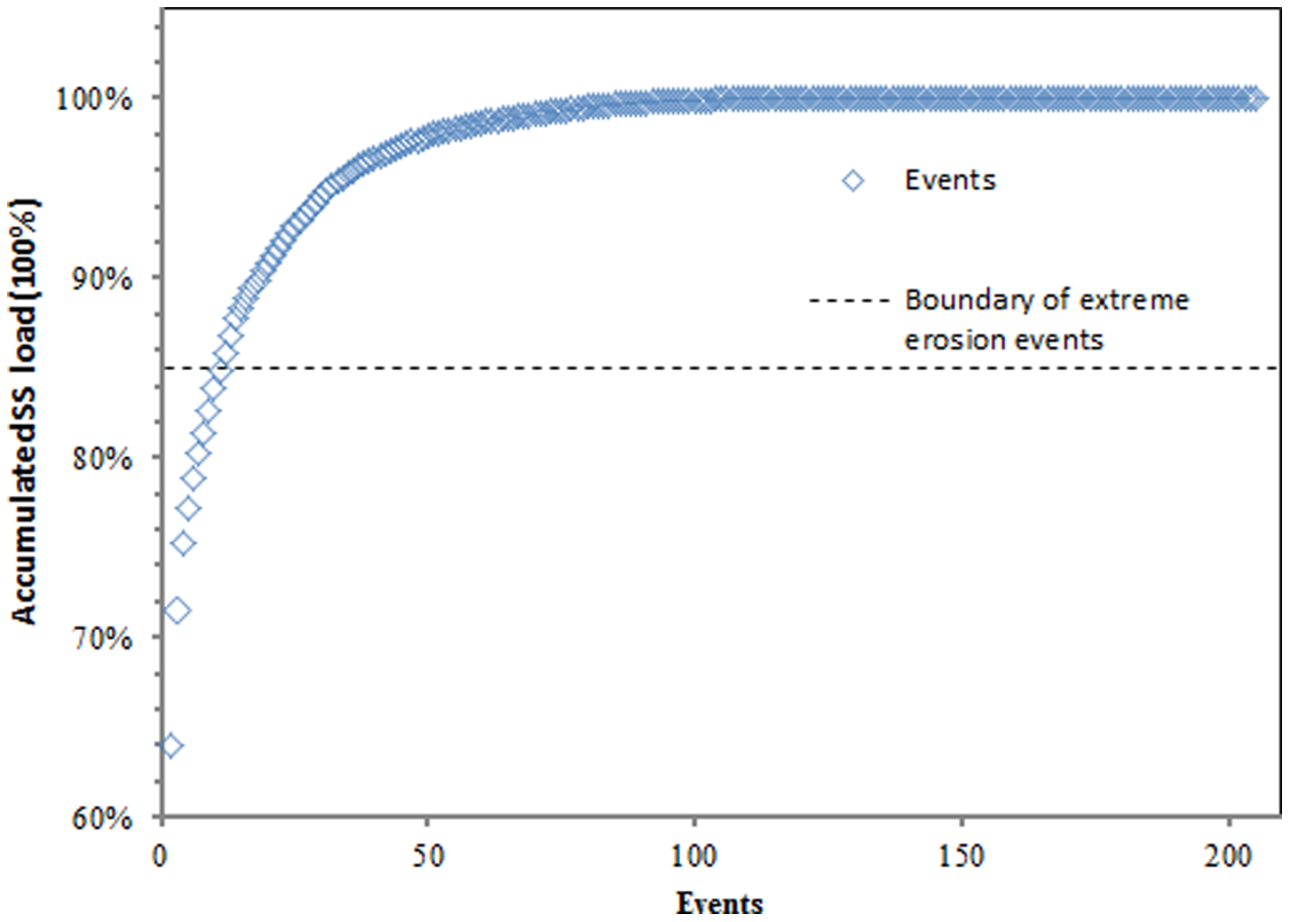
The percentage of accumulated suspended sediment yield in relation to the number of events. Events were independently ranked from largest to smallest for sediment.

**Figure 3:**
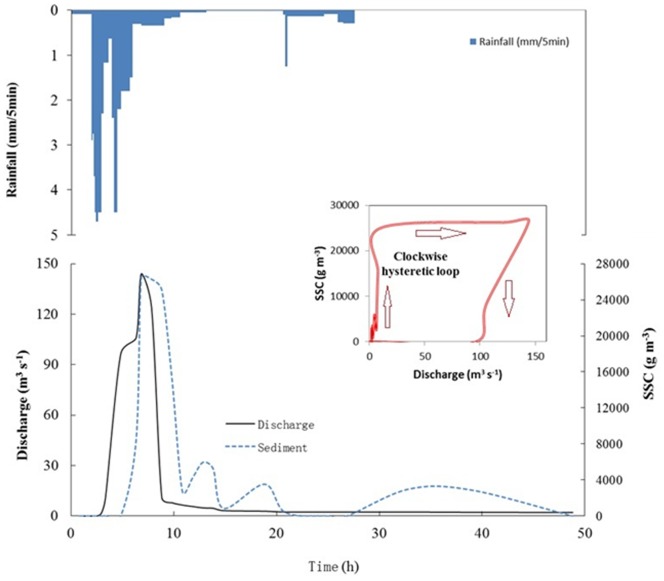
Hyetographs, hydrographs, sedigraphs, and hysteretic loop during the event of 6/7/1999.

The greatest proportion of the SS load was produced by the extreme erosion events. This finding is illustrated in Figure 2, which illustrates the percentage of the accumulated sediment load as a function of the percentage of events. Extreme erosion events were responsible for 83.8% of the *SS* loads. For each extreme erosion event, the erosive characteristics of individual storms were evaluated. The *I_30_* ranged from 6.7 to 24.7 mm h^−1^. Antecedent rainfall amounts were highly variable, ranging from 0 to 12.2, 0 to 33.2, and 0 to 15.8 mm of precipitation during the previous one-, five-, and seven-day periods, respectively. The maximum *SS* load was 33,987,157 kg on 7/6/1999, which was 4.2 times greater than the mean annual load during the study years (Fig. 3). This event was caused by a *Q_max_* of 144 m^3^/s. The curve representing sediment concentration vs. discharge over time exhibited a clockwise hysteretic loop [33]. The maximum *SS* occurred on 30/6/1991 and was 62,138 g/m^3^. This flood load was 2.68 times greater than the mean annual load and was generated by the highest precipitation recorded during the study period (P = 153.7 mm), which created a flood peak discharge of 87.9 m^3^ s^−1^ and a total flood runoff depth of 84.8 mm. The minimum *SS* load of an extreme erosion event was 1,033,247 kg. The *RC* of extreme erosion events ranged from 0.94 to 0.36. The peak discharge ranged from 16.2 to 144 m^3^ s^−1^. The direct *SSC_max_* ranged from 3,539 to 62,138 g m^−3^. *I_30_* ranged from 6.7 to 24.7 mm h^−1^. The antecedent rainfall amounts were highly variable, ranging from 0 to 12.2, 0 to 33.2, and 0 to 15.8 mm of precipitation during the previous one-, five-, and seven-day periods, respectively.

### Large Cumulative Rainfalls

**Figure 4:**
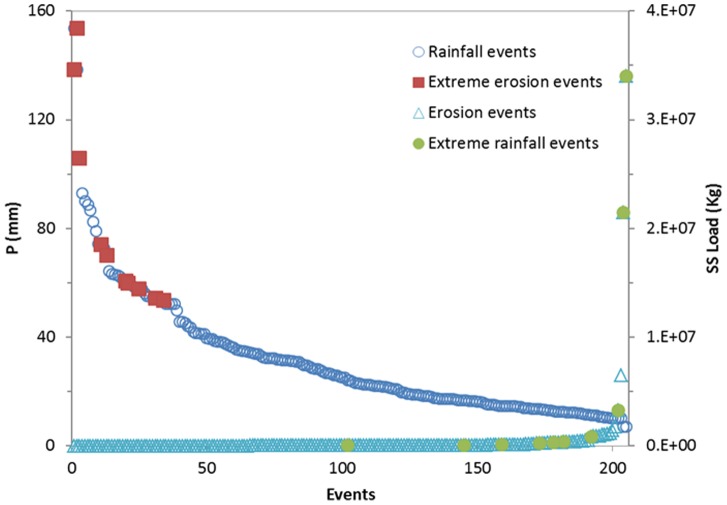
Distributions of extreme erosion events and extreme rainfall events.

The study of extreme erosion events is almost always associated with the study of precipitation [34]. To examine the relationship between rainfall and extreme erosion events, we identified the 10 events with the highest *P* values as large cumulative rainfalls (using the same method that was used to identify extreme erosion events). We then compared large cumulative rainfalls with extreme erosion events and evaluated the descriptive statistics of the extreme erosion and rainfall events, extreme erosion events, and large cumulative rainfalls shown in Table 3. Some studies have found that the most large cumulative rainfalls do not necessarily produce extreme fluvial discharge or the maximum soil erosion [7], [35], [36]. Our results confirm that the most extreme erosion events were not necessarily produced by the most large cumulative rainfalls. Only three extreme erosion events were found to be associated with large cumulative rainfalls. Fig. 3 illustrates the distributions of extreme erosion events and large cumulative rainfalls. A rainfall of 53.4 mm caused extreme erosion (117,504 kg) on 2/8/1998, whereas a rainfall of 74.3 mm caused only 53,428 kg of *SS* load on 9/4/1994. Extreme erosion events were greater than 16.6% if ranked by *P*, and large cumulative rainfalls were greater than 50% if ranked by *SS* load (Fig. 4).

**Table 3:** Characteristics of extreme erosion and rainfall (1), extreme erosion (2), and extreme rainfall events (3).

Date	Regime	D(min)	P(mm)	I30(mm)		SS (kg)	AP1d(mm)	AP3d(mm)	AP7d(mm)	RC	SSmax(g/m^3^)	Qmax(m^3^/s)
30/6/1991	1	973.2	153.7	20.1	21,467,789		1.2	1.2	10	0.55	62,138	87.9
6/7/1999	1	1,610	138.5	18.2	33,987,159		0	0	0	0.94	25,060	144
30/4/1990	1	2,890.2	105.9	18.9	3,224,476		0	0	8.9	0.5	42,820	61.2
1/8/1997	2	320	59.8	24.7	6,505,964		0	0	0	0.38	34,820	30.1
19/5/1993	2	1,669.8	54.3	14.9	1,738,339		7.1	8.6	28.4	0.52	40,745	19.6
22/7/1993	2	919.8	74	9.6	1,385,369		7.1	33.2	45.8	0.54	11,970	17.3
2/8/1998	2	1,400	53.4	6.7	1,175,040		12.2	23.3	34.7	0.54	2,289	24.1
28/6/1998	2	1,225	70.1	7.7	1,056,682		0	0	6.8	0.36	38,680	48.1
6/6/1996	2	2,820	57.7	8.2	1,033,247		2.9	65	71.8	0.84	3,539	16.2
6/7/1996	2	255	60.6	25.3	1,044,054		0	0	139.9	0.43	1,260	21.9
19/6/1990	3	1,596	92.8	8.9	329,701		0	0	0	0.39	3,007	11.9
25/7/1989	3	2,565	90	11.6	831,571		0.5	0.5	0.5	0.35	7,558	15.2
6/7/1995	3	3,400	88.8	6.4	7,483		0	2.1	2.1	0.25	1,030	3.08
2/8/1991	3	2,119.8	86.6	12.3	287,019		8	17.2	20.3	0.44	4,517	6.6
18/9/1996	3	2,345	82.3	15.9	215,205		35	35	37.8	0.39	1,333	14.3
12/6/2004	3	2,700	79	5.9	103,077		0.1	3.9	3.9	0.33	2,366	7.3
9/4/1994	3	610.2	74.3	9.1	53,428		0.2	2.7	8.4	0.1	5,070	5.6

### Canonical Discriminant Analysis

**Figure 5:**
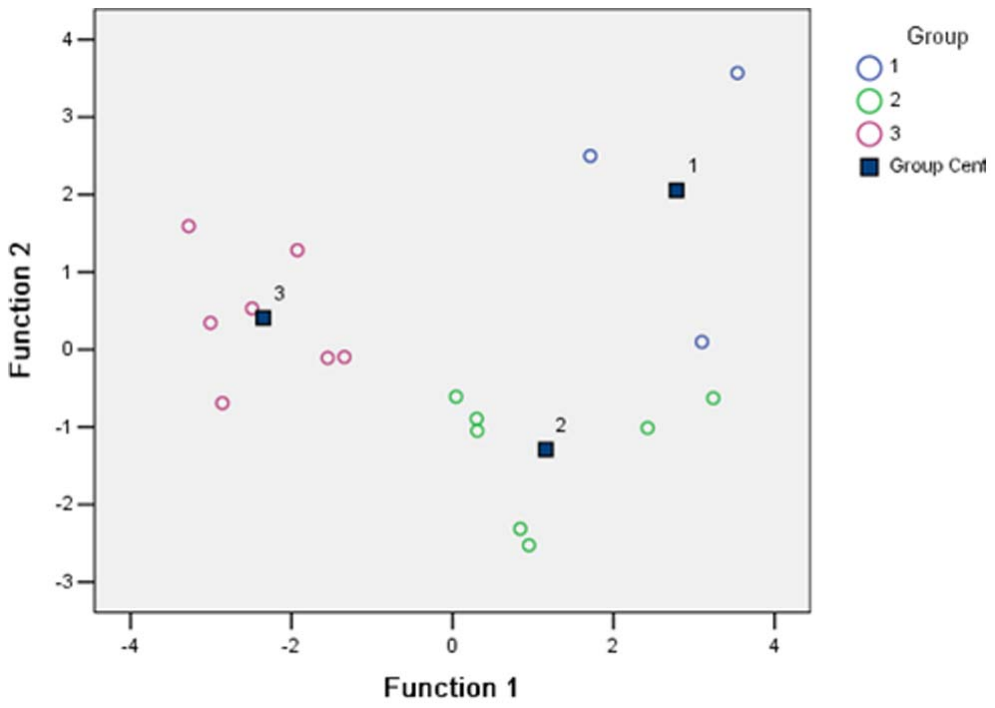
Distribution of cases with discriminant functions.

**Figure 6:**
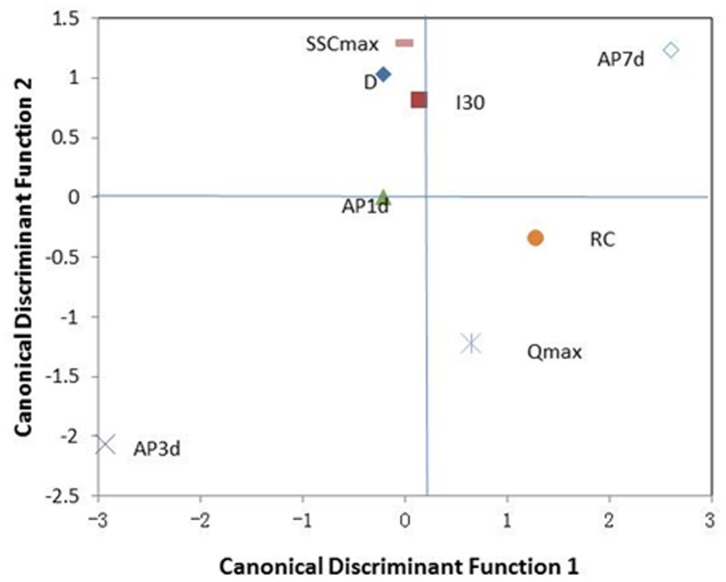
Standardized discriminant function coefficients of the variables included in the discriminant functions; the values are shown in **Table 3**.

To identify factors (excluded *TL* and *P*) that might explain the measured hydrological and sedimentological responses of extreme events, we conducted discriminant analyses using the data shown in Table 3. We created two canonical discriminant functions that included eight of the input variables to separate the groups (Table 4 and Fig. 5). The first canonical discriminant function (f1) explained 73.7% of the variance (Table 4), was highly positively correlated with *SSC_max_* (0.411), *RC* (0.409), and *Q_max_* (0.393), and was more weakly correlated with *I_30_* and *AP1d*. For this reason, f1 expresses increasing *SSC_max_*, *RC,* and *Q_max_* in the watershed at the time of the floods with higher function values. The second discriminant function (f2) correlated with the remaining parameters (−0.481 for AP7d and −0.417 for *SSC_max_*), as expected, given the lower proportion of variance explained. In addition, f2 was found to increase with increasing *SSC_max_*, although with low intensity. Table 3 also presents the discriminant function values at the centroids of the three event types. The distribution of the flood events in the two dimensions created by the functions is shown in Fig. 6 (discriminant function coefficients are shown in Table 4). The centroids of the extreme erosion and rainfall group were high for both functions (f1 = 2.783; f2 = 2.056). The centroids of the extreme erosion events had relatively small values for f2 but high values for f1. These events were generated for very high values of *RC*, *SSC_max_*, and *Q_max_*. The large cumulative rainfalls have very low values for f1 and values close to zero for f2 (0.408). That is, the large cumulative rainfalls were generated for particularly low values of *SSC_max_*, *RC*, and *Q_max_* but with no special antecedent rainfall characteristics.

**Table 4:** Data on the canonical discriminant functions (cdf).

Eigenvalues of cdf								Group centroids		
Eigenvalue	Variance (%)	Cumulative variance (%)				Canonical correlation		1	2	3
5.093	73.7	73.7				0.914		2.783	1.158	−2.351
1.819	26.3	100				0.803		2.056	−1.289	0.408
**Standardized cdf coefficients**										
	**D**		**I_30_**	**AP1d**	**AP3d**		**AP7d**	**RC**	**Qmax**	**SSCmax**
F1	−0.214		0.136	−0.217	−2.940		2.599	1.271	0.645	−0.084
F2	1.025		0.815	0.003	−2.068		1.235	−0.341	−1.220	1.289
**Structure matrix of cdf**										
	**SSC_max_**		**RC**	**Q_max_**	**I_30_**		**AP1d**	**AP7d**	**AP3d**	**D**
F1	0.411*		0.409*	0.393*	0.260*		−0.101*	0.111	0.041	−0.170
F2	0.272		0.054	0.004	0.143		−0.076	−0.481*	−0.417*	0.280*

The highest correlations of the variables with the functions are indicated with *.

## Discussion

**Figure 7:**
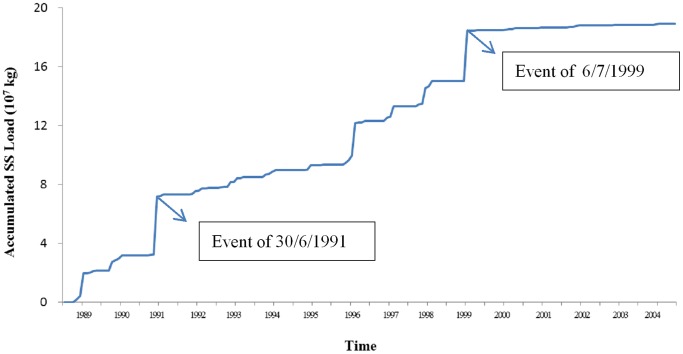
Curve of accumulated SS load during the study period. (The time step is one month.).

A comparison of the accumulated suspended sediment transport with the accumulated rainfall and the number of single events indicates that 83.3% of sediment transport occurred during 5% of the erosion events and was caused by 13% of the precipitation. Fig. 7 illustrates the importance of the SS loads of extreme events to the total accumulated SS load. Two sharp rises in the curve were caused by the events of 1991/06/30 and 1999/07/06. The soil thickness of the Wangjiaqiao watershed ranges predominantly from 30 to 50 cm. The most extreme erosion event caused a lot of soil loss in the watershed. In the TGA, the sediment delivery rate on a small watershed scale has been thought to be very low in “common events,” ranging from 0.1 to 0.4 [37], [38], but mean sediment delivery radios are inappropriate for characterizing extreme erosion events. A study by Zhang et al. [39] reported a long-term natural sediment delivery radios close to 1 for a 10.88-km^2^ watershed.

The results confirm the complexity of erosion processes and the relationships between *TL* and *P* in particular. The most cumulative rainfalls do not necessarily produce the most extreme erosion. Events of low rainfall depth and short duration typically cause very limited hydrological responses and almost no sediment transport. Other studies [17], [40] have suggested that sediment yield rates may change in response to changes in rainfall, including changes in both total precipitation and intensity.

The geomorphologic impacts of an extreme event are closely related to such factors as the erodibility of the parent material, topography, vegetation cover, and land use [41]–[43]. Moreover, soil erosion is largely determined by on-site sediment production and the connectivity of sediment sources and streams [44]. Lu and Higgitt [45] found that 60% of sediment is contributed from arable land in 32 catchments in the TGA. Slopes with gradients in excess of 30% comprise 76% of the area of the Wangjiaqiao watershed. Cultivated sloping lands are major contributors to sediment yield. Tillage activities are generally carried out between April and September. Soil is eroded and then transported to the stream networks [27]. The soil parent material of the watershed is predominantly purple sandy shale, and bedrock is typically exposed in the channel bed; thus, channel erosion is rare. Sediments stored in the channel and distributed within tributaries are transported after flood events with sufficient transport capacity [46]. Therefore, the extreme erosion events characterized by the largest values of *SSC_max_*, *RC*, and *Q_max_* could be understood to result from the transport of deposited sediment within the stream bed during previous events. The clockwise hysteretic loop of the largest erosion event confirms this explanation [47]. Moreover, bank collapse has been identified as an important source of extreme erosion. Investigations in the Wangjiaqiao watershed have indicated that bank collapse along the stream results in bank erosion that contributes to sediment transport, particularly during major floods of high intensity [48]. The soils of the watershed are mainly purple soils developed from purple sandstone with a content of rock fragments and are rich in macrospores [49]. The macrospores of shallow soils become saturated easily during large rainfall events, the soil bulk density increases rapidly, and the combination of poor drainage in the soil and high-intensity rainfall easily causes bank collapse [50].

## Conclusions

This study investigated the important issue of extreme erosion events in a typical watershed in the TGA. These events, which occur primarily during the summer season, can result in serious soil loss and great damage. The results confirm the complex and heterogeneous nature of hydrological and sediment responses in the watershed. A total of 83.3% of the sediment transport occurred during 5% of the erosion events that occurred in the TGA and was caused by 13% of the precipitation that the TGA received. The maximum TL of an event was 4.2 times the mean annual load during the study period. Large rainfall depth does not necessarily produce large fluvial discharge or large soil erosion. Extreme erosion events were generated under very high *RC*, *SSC_max_*, and *Q_max_*. The results indicate that the use of event average values or mean index values may not be suitable for analyzing soil erosion processes in the TGA. The effects of a small number of events appear to determine the magnitudes of soil loss; catastrophism may be more suitable for explaining soil erosion processes. Although our work provides a considerable database of extreme sediment load during rainfall events, we provide a speculative explanation concerning extreme erosion events, subject to the constraints of the field methods. Further investigations should be performed to assess the sediment yield during extreme events, which is expected to be of significant value in environmental management and development of strategies to control sediment dynamics at the catchment scale.
